# Responses of Vaginal Microbiota to Dietary Supplementation with Lysozyme and its Relationship with Rectal Microbiota and Sow Performance from Late Gestation to Early Lactation

**DOI:** 10.3390/ani11030593

**Published:** 2021-02-24

**Authors:** Shengyu Xu, Yanpeng Dong, Jiankai Shi, Zimei Li, Lianqiang Che, Yan Lin, Jian Li, Bin Feng, Zhengfeng Fang, Zhuo Yong, Jianping Wang, De Wu

**Affiliations:** 1 Animal Nutrition Institute, Sichuan Agricultural University, Chengdu 611130, Sichuan, China; dongyp1995@163.com (Y.D.); shijiankai0227@sina.com (J.S.); 13778194073@163.com (Z.L.); clianqiang@hotmail.com (L.C.); able588@163.com (Y.L.); lijian522@hotmail.com (J.L.); fengb123d@163.com (B.F.); fangzhengfeng@hotmail.com (Z.F.); zhuoyong@sicau.edu.cn (Z.Y.); wangjianping1983@hotmail.com (J.W.); wude@sicau.edu.cn (D.W.); 2 Key laboratory of Animal Disease-Resistant Nutrition, Ministry of Education, Ministry of Agriculture and Rural Affairs, Chengdu 611130, Sichuan, China

**Keywords:** lysozyme, sow, vaginal microbiota, metabolites, late gestation and early lactation

## Abstract

**Simple Summary:**

The vaginal microbiota has a crucial role for the health of the sow and the newborn piglet. The purpose of this study was to investigate the effect of dietary supplementation with lysozyme in the vaginal microbiota and evaluate its relationship with the fecal microbiota of the rectum and the reproductive performance of the sow. The results suggest that, lysozyme supplementation changed vaginal microbiota composition at different taxonomic levels, the changed vaginal microbiota was associated with variations in fecal microbiota, and these changes correlated with some reproductive performance of the sow.

**Abstract:**

This study was conducted to evaluate the effects of dietary lysozyme (LZM) supplementation on the vaginal microbiota, as well as the relationship between vaginal microbiota and the fecal microbiota of rectum and the reproductive performance of the sow. A total of 60 Yorkshire × Landrace sows (3–6 of parity) were arranged from day 85 of gestation to the end of lactation in a completely randomized design with three treatments (control diet, control diet + lysozyme 150 mg/kg, control diet + lysozyme 300 mg/kg). The results showed that sows fed with lysozyme increased serum interleukin-10 (IL-10, *p* < 0.05) on day 7 of lactation. The vaginal microbiota varied at different taxonomic levels with LZM supplementation by 16S rRNA gene sequencing. The most representative changes included a decrease in Tenericutes, *Streptococcus*, *Bacillus* and increase in Bacteroidetes, Actinobacteria, *Enterococcus*, and *Lactobacillus* (*p* < 0.05). There were 777 OTUs existing in both, vaginal and fecal microbiota. The addition of LZM also decreased the abundance of Tenericutes (*p* < 0.05) in the vagina and feces. The changes in the microbiota were correlated in some cases positively with the performance of the sow, for example, *Bacillus* in feces was positively correlated with the neonatal weight (*p* < 0.05). These results indicate that the addition of lysozyme to the diet of sow during perinatal period promote the change of vaginal bacterial community after farrowing. The variations in vaginal microbiota are also associated with the changes in the fecal microbiology of the rectum and the reproductive performance of the sow. Therefore, it is concluded that dietary supplementation with lysozyme in sows in late gestation stage until early lactation, is beneficial to establish vaginal microbiota that seems to promote maternal health and reproductive performance.

## 1. Introduction

Lysozyme (LZM) is a naturally occurring antimicrobial enzyme found in the mucosal barrier of all mammals [1]. This enzyme is used as an additive in infant food and in the medical industry due to its anti-infective nature [1,2,3]. LZM is a 1,4-β-N-acetylmuramidase, which can cleave the β-1,4-glycosidic bond between the N-acetylmuramic acid and N-acetylglucosamine residues of the bacterial peptidoglycan. So LZM can cause an incomplete cell membrane and lead to cell death of bacterium [4]. Hydrolysis of products produced from the loss of the bacterium cellular membrane stimulate immunoglobulin A (IgA) secretion, macrophage activation, and the rapid clearance of bacterial pathogens in the organism [5]. This suggests that dietary LZM would lead to variation in the gut microbiota and cytokines, which has been confirmed in piglets [6,7]. Thus, it has been attempted to use as an alternative to antibiotics in husbandry [8,9,10,11]. Cytokines such as IL-1β and TNF-α are known to mediate and have function in the inflammatory response [12]. Adding LZM to pigs’ diets decreased the TNF-α concentration [13]. In a colitis porcine model, lysozyme was observed to up-regulate the mRNA abundance of the anti-inflammatory cytokines IL-4 and TGF-β [14]. Gut microbiota plays a crucial role in the metabolism of nutrient, immune function, gut hormone secretion and provide protection from pathogens [15,16]. Simultaneously, gut microbiota is regulated by nutrient as well as the immunological and metabolic statuses of the animal [17]. In our previous study, with the dietary lysozyme supplementation in sow during late gestation to lactation the fecal bacteria changed [18], the average daily feed intake of lactation stage increased and the weaning-to-estrus interval decreased [19]. In addition to the close relationship between intestinal bacteria and sows’ reproductive performance, vaginal bacteria are also extremely important to the health and reproductive performance of sow.

Several studies reported that the production of lactic acid, bacteriocins, hydrogen peroxide, etc. by the vaginal microbiota, plays an important role in the health of the maternal reproductive tract [20,21]. Initial microbiota colonization of a piglet’s microbiota occurs during birth after exposure to the sow’s vaginal, fecal and cutaneous microorganism [22]. In contrast to the growing number of studies characterizing the intestinal or fecal microbiome of pigs and humans [17,23,24,25]. There are only few studies available that characterize the vaginal microbiome and its development in pregnant and lactating sows [26,27]. In humans, a decrease in lysozyme levels was found in the vaginal mucosa barrier associated with an increase in vaginal bacterial diversity [28]. However, how lysozyme supplementation affects the vaginal microbiota of sows is still unknown. Furthermore, the vaginal microbiota, as well as its changes with farrowing and lactation, is not well explored.

Therefore, the objectives of this study were to investigate the effects of dietary supplementation with lysozyme on the vaginal microbiota of sows in the final stage of gestation until the beginning of lactation, as well as the relationship between the vaginal and rectum fecal microbiota and reproductive performance.

## 2. Materials and Methods

All animal procedures used in this study were approved by the Animal Experimental Committee of Sichuan Agricultural University (Ethic Approval Number DKY-S20156137).

Lysozyme 5000 U/mg, supplied by Shanghai Longyou Biotechnology Co, Ltd., Shanghai, China.

### 2.1. Animals and Experimental Design

Sixty pregnant sows (day 85, Yorkshire × Landrace; 3–6 parity) were randomly allocated to three treatments as follows: control (basal diet, n = 20), LZMA (basal diet + 150 mg/kg of lysozyme, n = 20), and LZMB (basal diet + 300 mg/kg of lysozyme, n = 20). This experiment was carried out with the same batch of sows at all timepoints as previous studies [18,19]. Sows were supplemented with lysozyme from day 85 of gestation to day 21 of lactation, when piglets were weaned. The basal diet used was the same as in the previous study [19]. This basal gestation diet contained 3.04 Mcal of digestible energy per kilogram (DE/kg), 14.65% crude protein, 0.69% Lys, 0.85% calcium, and 0.67% phosphorus. While the basal lactation diet contained 3.29 Mcal DE/kg, 17.54% crude protein, 0.99% Lys, 0.99% calcium and 0.68% phosphorus. No antibiotics, probiotics, or other medications were used during the study.

Sows were housed in individual gestation stalls prior to day 106 of gestation and were transferred to individual farrowing crates at day 107 of gestation. Sows were fed an average diet of 3.5 kg/d and two times/d during the late gestation stage, fed 0.5 kg of diet on the day of farrowing, and then gradually increased by 1.0 kg/d and two times/d up to the maximum amount of feeding. During the lactation days, free access to feed and water was maintained.

### 2.2. Sample Collection

On the farrowing day (d0) and day 7 of lactation (d7), 10 mL blood was sampling from the ear vein of eight sows (same batch of sows used in Xu et al. 2018 for blood samples [19]) per treatment after an overnight fast (12 h). The 8 sows were randomly selected from the 10 sows, which were used in Xu et al. 2020 for metabolic biomarkers of fecal samples [18]. Blood samples were centrifuged at 4 °C, 3000× *g* for 15 min to obtain the serum which was stored at −20 °C for further analysis. We randomly selected 6 sows (same batch of sows used in Xu et al. 2020 for fecal bacterial community analysis [18]) from the 8 sows which were used for the blood sampling, and we collected the vaginal samples. A swab method was used to obtain the vaginal contents. Each swab was immediately placed in a sterile 5 mL screw cap tube, which was prefilled with 2 mL of phosphate-buffered saline (PBS). The vaginal samples were kept in liquid nitrogen, and then transferred to −80 °C to store until DNA extraction.

### 2.3. Serum Analyses

Blood samples from the sows were analyzed by enzyme-linked immunosorbent assay (ELISA) Kits (Jiancheng Institute of Biological Technology, Nanjing, China; porcine specific antibodies) for the interleukin-6 (IL-6), interleukin-10 (IL-10) and tumor necrosis factor-α (TNF-α). The cytokine analysis was performed according to the manufacturer’s instructions. The minimal detection limit for IL-6, IL-10, and TNF-α were 12.5 ng/l, 5 ng/l and 7 ng/l, respectively.

### 2.4. Bacterial Community Analysis

The microbial DNA of vaginal sample was extracted using the Mo Bio PowerFecal^TM^ DNA Isolation Kit (MO BIO Laboratories, Carlsbad, CA, USA). Nucleic acid/protein analyzer (Beckman DU-800, Beckman Coulter, Inc., CA, USA) was used to determine the concentration and purity of DNA. The DNA samples were sent to a commercial service provider (Novogene Bioinformatics Technology, Beijing, China) for paired-end sequencing on Illumina HiSeq PE250 platforms and bioinformatics analyses. Using a forward primer 515f (5′-GTGCCAGCMGCCGCGGTAA-3′) and a reverse primer 806r (5′-GGACTACHVGGGTWTCTAAT-3′) to amplify the V4 hypervariable region of the 16S rRNA gene as described as before [18].

High-quality tags were filtered according to Xu et al. [18], and clustered into OTUs utilizing Uparse v7.0.1001 (http://drive5.com/uparse/) at 97% sequence similarity. The Ribosomal Database Project (RDP) classifier Version 2.2 (http://github.com/rdpstaff/) was applied to assign taxonomy for 16S rRNA gene sequences. Annotated the representative sequence of OTUs. The Mothur method and the SSUrRNA database of SILVA (http://www.arb-silva.de/) were used to perform species annotation analysis (with a threshold of 0.8–1) to obtain taxonomic information. Venn diagram was generated for comparison among the OTUs of the treatments. For vaginal microbiota alpha diversity values for each sample were assessed by Qiime 1.7.0. For vaginal microbiota beta diversity analysis, the relationship in vagina microbiome among the treatments were examined by principal coordinate analysis (PCoA) based on binary jaccard distances.

### 2.5. Statistical Analysis

Data of relative abundance at the phylum and genus level in vaginal sample were log-transformed before statistical analysis. The data were analyzed using the General Linear Model (GLM) procedures of SAS (V9.3, SAS Institute Inc., Cary, NC, USA) followed by a DUNCAN analysis for multiple comparison when the F test in the analysis of variance table was significant for the different treatment. The serum cytokines of sow, relative abundances at phyla and genera level were analyzed using the following statistical model: *Y_ij_ = μ + t_i_ + e_ij_* where *Y_ij_* is the analyzed variable, *μ* is the overall mean, *t* is the effect of treatment (*_i_* = 1, 2, 3), and *e* is the residual error (*_i_* = 1, 2, 3, *_j_*= 1 … 8 or 6). A paired *t*-test was used to detect the differences between the two time point in the same treatment (Table 1 and Table 2). The vaginal microbiota alpha diversity index was analyzed by MIXED procedure of SAS, according to the following model: *Y_ijk_ = μ + α_i_ + β_j_ + (αβ)_ij_ + e_ijk_*, in which Y is the analyzed variable, μ is the overall mean, *α_i_* is the effect of treatments (*_i_* = 1, 2, or 3), *β_j_* is the effect of lactation time (*_j_* = 1 or 2), (αβ)*_ij_* refers to the interaction between treatments and lactation time, *e_ijk_*; represents the residual error (Table 3). Data were corrected by false discovery rate analysis according to the Benjamini–Hochberg method with an α of < 0.05 in all treatments (Table 1 and Table 3 and Supplementary Table S2) and genera (Table 3, Supplementary Table S2) [29]. All data were expressed as means ± standard deviation (SD). Differences were considered significant at *p* < 0.05, whereas 0.05 < *p* < 0.10 was considered as a tendency.

Correlations between vaginal microbiota and metabolic parameters in serum, vaginal or rectal microbiota and the sow reproductive performance, where analyzed by Spearman’s correlation in R 3.0.2 with the Rstudio 0.97.310 package, and heat map was generated using gplots R package. The rectual microbiota and reproductive performance data come from previous studies [18,19]. Differences of *p* < 0.05 were considered significant, whereas *p* < 0.10 was considered a tendency.

## 3. Results

### 3.1. Effect of Lysozyme Diet Supplementation on Serum Cytokines of Sow

Sows fed with 150 mg/kg and 300 mg/kg lysozyme diets enhanced (*p* = 0.02) the serum concentration of IL-10 on day 7 of lactation (Table 1) compared with control. Sows fed with 150 mg/kg lysozyme diets had a tendency to increase serum concentration of IL-10 (*p* = 0.08) on the day of farrowing.

### 3.2. Effect of Lysozyme Diet Supplementation on Sows’ Vagina Microbial Diversity

**Figure 1:**
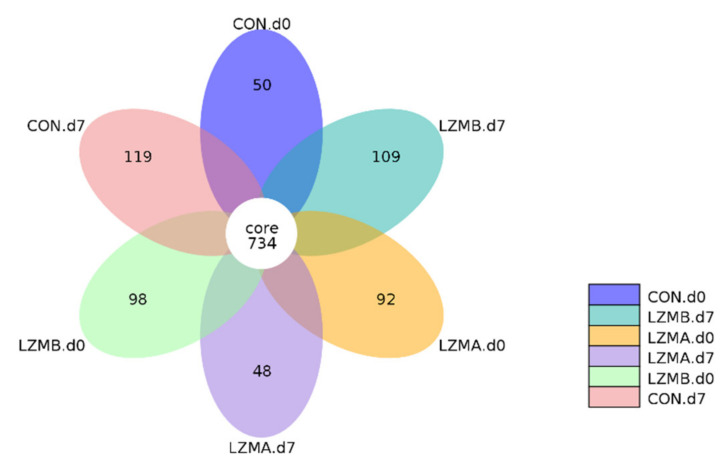
Venn diagrams were generated to compare OTUs between the different treatments of sow vaginal samples on the day of farrowing (d 0) and day 7 of lactation (d7). Venn diagram was generated to describe the common and unique OTUs among treatments at different day of lactation in sow vagina. CON. d0 = control diet at the day of farrowing, CON. d7 = control diet at day 7 of lactation, LZMA d0 = control diet + lysozyme 150 mg/kg at the day of farrowing, LZMA d7 = control diet + lysozyme 150 mg/kg at day 7 of lactation, LZMB d0 = control diet + lysozyme 300 mg/kg at the day of farrowing, LZMB d7 = control diet + lysozyme 300 mg/kg at day 7 of lactation.

**Figure 2:**
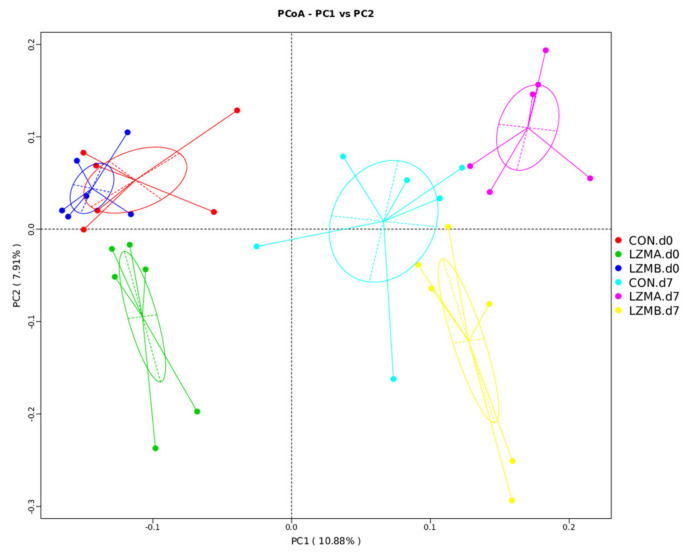
Comparison of the vaginal microbiota composition among treatments. Principal coordinate analysis to visualize the binary jaccard distances of vagina samples from individual sow. CON. d0 = control diet at the day of farrowing, CON. d7 = control diet at day 7 of lactation, LZMA d0 = control diet + lysozyme 150 mg/kg at the day of farrowing, LZMA d7 = control diet + lysozyme 150 mg/kg at day 7 of lactation, LZMB d0 = control diet + lysozyme 300 mg/kg at the day of farrowing, LZMB d7 = control diet + lysozyme 300 mg/kg at day 7 of lactation.

A total of 36 vaginal samples were subjected to 16S rRNA gene sequencing. Supplementary Table S1 showed the raw reads, effective tags and operational taxonomic units (OTUs) average for each treatment. A set of 734 OTUs existed in all treatments and were thus defined as core OTUs (Figure 1), which comprised 84.2% of the total number of OTUs. The alpha and beta diversity of the vaginal microbiota were evaluated to determine the bacterial diversity. LZMA and LZMB treatments reduced the observed species and Chao 1 index (richness) at day 7 of lactation (*p* < 0.05, Table 3). The observed species and Chao 1 index on the farrowing day were higher than day 7 of lactation in all treatments (*p* < 0.05). For beta diversity analysis, the vaginal microbiota distribution of CON. d0, LZMA. d0, CON. d7, LZMA d7 and LZMB d7 was distinctly clustered separately, as shown in Figure 2, examined by principal coordinate analysis.

### 3.3. Changes of Vaginal Microbiota Composition by Lysozyme Supplementation in Sow

**Figure 3:**
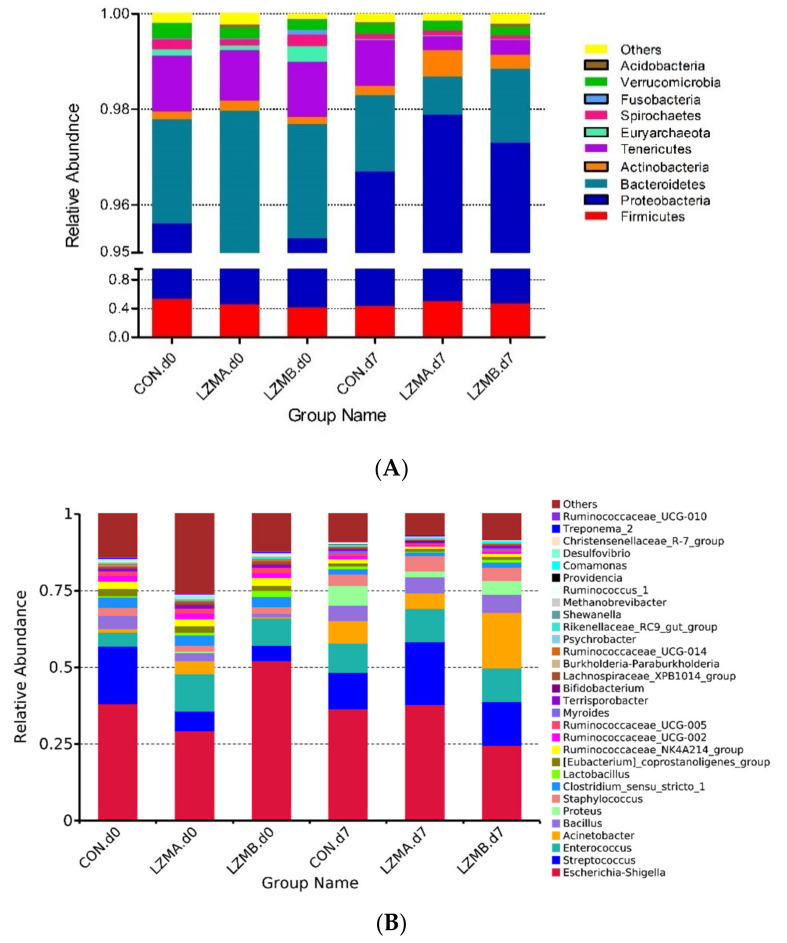
16S rRNA gene analysis reveals phyla (**A**) and genus (**B**) level differences in sow vagina between the treatments. CON. d0 = control diet at the day of farrowing, CON. d7 = control diet at day 7 of lactation, LZMA d0 = control diet + lysozyme 150 mg/kg at the day of farrowing, LZMA d7 = control diet + lysozyme 150 mg/kg at day 7 of lactation, LZMB d0 = control diet + lysozyme 300 mg/kg at the day of farrowing, LZMB d7 = control diet + lysozyme 300 mg/kg at day 7 of lactation.

The effects of lysozyme on the relative abundance at phyla and genus level of the vaginal microbiota are displayed in Figure 3. The top six dominated phyla are Proteobacteria, Firmicutes, Bacteroidetes, Actinobacteria, Tenericutes and Euryarchaeota, as shown in Figure 3A. Proteobacteria, Firmicutes and Bacteroidetes are the most abundant (accounted for more than 98.1%). The relative abundances of dominated genera (>0.1%) are presented in Figure 3B. Furthermore, the phyla (>0.1%) and genera (>0.3%) were chosen for significance analyses. The LZM treatments enhanced (*p* < 0.05) the relative abundance of Bacteroidetes on the farrowing day, on the other hand reduced Tenericutes (*p* < 0.05) on day 7 of lactation at phyla level, as shown in Table 2. Euryarchaeota decreased with the lactation progress as well as Bacteroidetes and Tenericutes in LZM treatments.

At genera level, 16 genera relative abundances changed across the different treatments on the farrowing day and day 7 of lactation (Table 2). Sows which had been fed with LZMA and LZMB diets had reduced relative abundances of *Streptococcus*, *Bacillus*, *Oscillospira* and *Family_XIII_AD3011_group* (FDR *p* value = 0.052) on the farrowing day, reduced relative levels of *Ruminococcaceae_UCG-002*, *Ruminococcaceae_UCG-005* and *Family_XIII_AD3011_group* on day 7 of lactation, and enhanced relative abundances of *Enterococcus* and *Acinetobacter* on the farrowing day. However, LZMA treatment had reduced *Burkholderia-Paraburkholderia* on the farrowing day, and reduced *Ruminococcaceae_NK4A214_group* and *Terrisporobacter*, enhanced relative abundance of *Streptococcus* compared with control treatment on day 7 of lactation. LZMB treatment had reduced relative abundance of *Escherichia-Shigella* on day 7 of lactation, enhanced relative abundance of *Lactobacillus*, *Lachnospiraceae_AC2044_group* on the farrowing day and *Acinetobacter* on day 7 of lactation. The relative abundances of *Ruminococcaceae_NK4A214_group*, *Ruminococcaceae_UCG-002*, *Ruminococcaceae_UCG-005*, *Ruminococcaceae_UCG-010* (Supplementary Table S2), *Oscillospira* and *Family_XIII_AD3011_group* were decreased with the lactation progress.

### 3.4. Correlations between the Vaginal Microbiota and Cytokines in Sow

In vaginal microbiota, at the phylum level, Tenericutes and Deinococcus.Thermus were negatively correlated with serum IL-10 (r = −0.48, *p* < 0.01; r = −0.36, *p* = 0.03; Figure S1). However, Cyanobacteria was positively correlated with serum IL-10 (r = 0.42, *p* = 0.01). Planctomycetes and Euryarchaeota were negatively correlated with serum IL-6 (r = −0.49, *p* < 0.01; r = −0.44, *p* < 0.01). Elusimicrobia and Synergistetes were negatively correlated with serum TNF-α (r = −0.34, *p* = 0.05; r = −0.36, *p* = 0.03).

**Figure 4:**
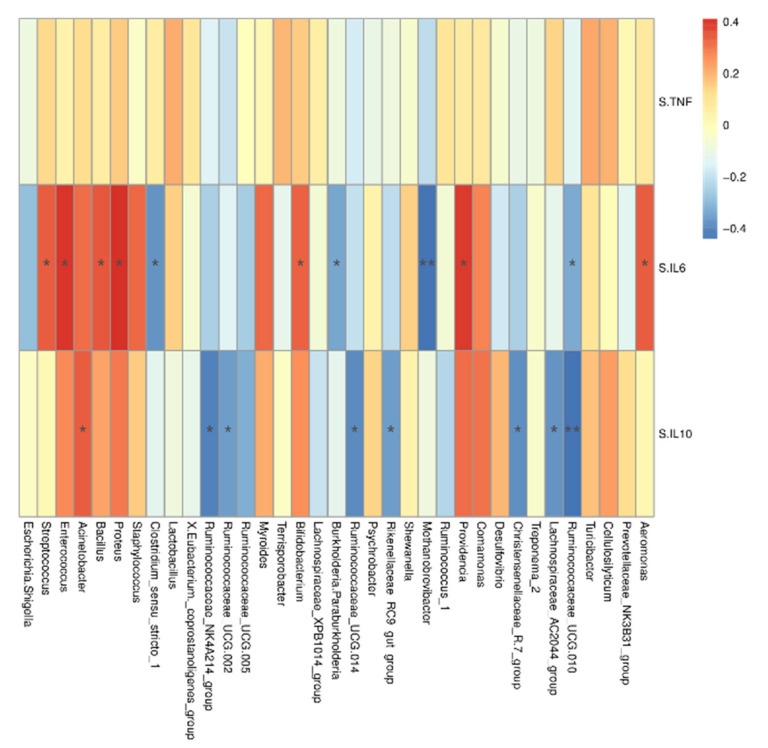
Heatmap of the spearman r correlations between the vaginal microbiota (genera level) significantly modified by metabolic parameters of sow. Data are presented as means ± SD (n = 6). * *p* < 0.05; ** *p* < 0.01 (following the Spearman correlation analysis). S.TNF = serum TNF-α, S.IL6 = serum IL-6, S.IL10 = serum IL-10.

Correlation analysis of vaginal microbiota at the genus level is shown in Figure 4, Ruminococcaceae_UCG.010, Lachnospiraceae_AC2044_group, Christensenellaceae_R.7_group, Rikenellaceae_RC9_gut_group, Ruminococcaceae_UCG.014, Ruminococcaceae_UCG.002 and Ruminococcaceae_NK4A214_group were negatively correlated with serum IL-10 (r ≤ −0.36, *p* < 0.05). However, Acinetobacter was positively correlated with serum IL-10 (r = 0.36, *p* = 0.04). Ruminococcaceae_UCG.010, Methanobrevibacter, Burkholderia. Paraburkholderia and Clostridium_sensu_stricto_1 were negatively correlated with serum IL-6 (r ≤ −0.33, *p* < 0.05). Aeromonas, Providencia, Bifidobacterium, Proteus, Bacillus, Enterococcus and Streptococcus were positively correlated with serum IL-6 (r ≥ 0.33, *p* < 0.05).

### 3.5. Relationship between the Vaginal or Rectum Microbiota and the Sow Performance

**Figure 5:**
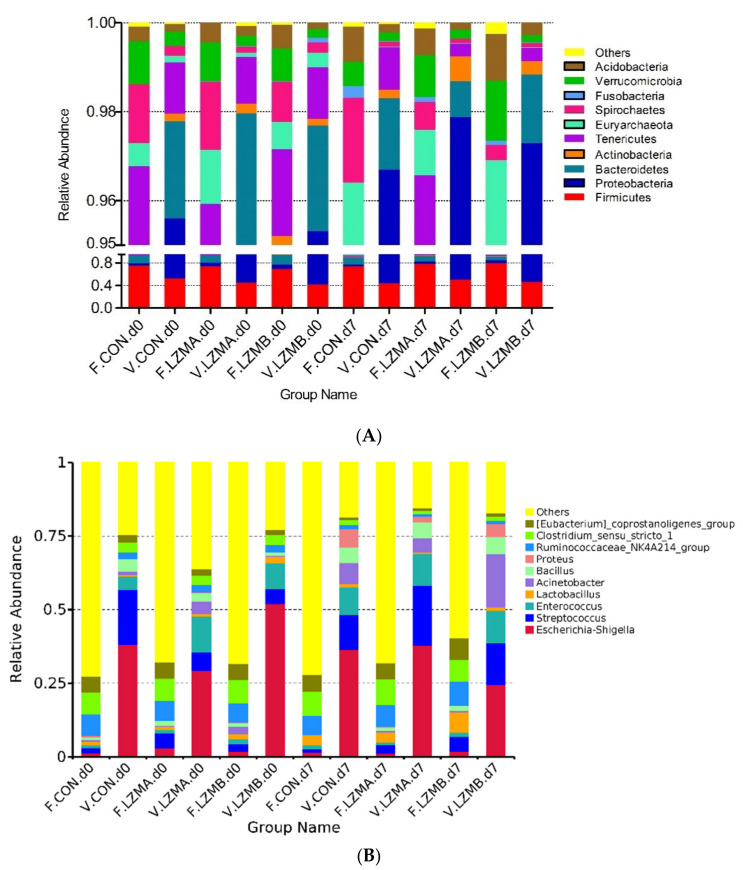
16S rRNA gene analysis reveals phyla (**A**) and genus (**B**) level differences between vaginal and rectum microbiota, and the treatments. F. = sample from fecal, V. = sample from vagina, CON.d0 = control diet at the day of farrowing, CON.d7 = control diet at day 7 of lactation, LZMA d0 = control diet + lysozyme 150 mg/kg at the day of farrowing, LZMA d7 = control diet + lysozyme 150 mg/kg at day 7 of lactation, LZMB d0 = control diet + lysozyme 300 mg/kg at the day of farrowing, LZMB d7 = control diet + lysozyme 300 mg/kg at day 7 of lactation.

There were 777 OTUs existed in all treatments compared with the vaginal and fecal microbiota (Figure S2), which comprised 84.2% of the total number of OTUs. As shown in Figure 5, the mainly three abundant phyla Firmicutes, Proteobacteria and Bacteroidetes accounted for 92.5% of microbiota in the feces (90.4–94.6%) vs. 98.1% in the vagina (97.5–98.7%). Proteobacteria abundance was higher in the vagina than the rectum (*p* < 0.01, 49.7% vs. 5.2%). However, the abundance of Bacteroidetes was lower in the vagina than the rectum (*p* < 0.05, 1.91% vs. 12.5%). At genus level, the abundance of *Escherichia-Shigella*, *Streptococcus* and *Enterococcus* were higher in the vagina than the rectum (*p* < 0.05, 33.81% vs. 1.83%; 14.92% vs. 3.08%; 8.54% vs. 1.57%). However, the abundance of *Lactobacillus* tended to be lower in the vagina than the rectum (*p* < 0.1, 0.96% vs. 2.92%).

As shown in Table 4, the relationships between microbiota of vagina or rectum at genus level and total born piglets, number of piglets born alive, stillborn and neonatal weight are presented. The relative abundances of *Desulfovibrio* in vagina and feces of sows were positively correlated with number of piglets born alive (*p* < 0.1 or *p* < 0.01). The relative abundances of *Terrisporobacter* in sows’ feces were positively corrected with number of piglets born alive (*p* < 0.05). However, the relative abundances of *Clostridium_sensu_stricto_1* and *Ruminococcaceae_UCG.014* in sows’ feces were negatively corrected with the number of piglets born alive (*p* < 0.05). The relative abundances of *Bacillus* in sows’ feces were positively corrected with neonatal weight (*p* < 0.05).

## 4. Discussion

This study investigates the effect of dietary supplementation with lysozyme on the sow’s vagina microbiota community structure and composition. Although adding lysozyme didn’t change the lysozyme concentration in the sows’ serum and milk (data not shown), lysozyme diet increased the serum anti-inflammatory cytokine IL-10 and varied the vagina microbiota diversity and composition. As shown in the principal coordinate analysis, the microbiota distribution of control and LZMA (150 mg/kg lysozyme diets) on the farrowing day, control, LZMA and LZMB (300 mg/kg lysozyme diets) on the day 7 of lactation were distinctly clustered separately. These indicated that lysozyme affected the vaginal bacterial community structure. Interestingly, it was also found that the vaginal microbiota community richness was decreased with the progress of lactation, opposite to the fecal microbiota community richness discussed in previous studies [18,30]. In a previous study the lowest gut microbiota richness was found on the farrowing day compared with day 7 and 21 of lactation [18], and Cheng et al. found that the lowest fecal microbial richness on day 3 of lactation [30]. From these results, it can be seen that the vaginal microbiota and the gut microbiota change in completely different ways with the advancement of the physiological stage from gestation to lactation. The present study results may help understand how the abundance of maternal vaginal microbiota changes with the physiological process of farrowing, as well as how microbiota continue to remodel and stabilize over the time in sow vagina.

It has been reported that Firmicutes are the main component of vaginal microbes in healthy sows, followed by Proteobacteria and Bacteroidetes [26]. The abundant phyla Firmicutes and Bacteroidetes accounted for 92% of microbiota in the sow vagina in this study, which is in agreement with previous study in pigs [27]. Bacterial phyla Fusobacteria, Proteobacteria, and Bacteriodetes in vagina are associated with postpartum fever and uterine diseases (such as: metritis and endometritis) identified in previous studies in cows and pigs [26,31,32]. Although bacterial species within the phyla Fusobacteria and Bacteroidetes are commonly associated with bovine necrotic vulvovaginitis and human bacterial vaginosis [26,33]. It has been reported that healthy and endometritis sows showed differences at the phylum level of Firmicutes, Proteobacteria and Bacteroidetes [26]. Although LZMA increased the abundance of Bacteroidetes, no sows showed endometritis in this study. The Tenericutes decrease with the dietary lysozyme supplementation was similar to our previous studies [18] and Everard’s [34] results. Everard et al. 2014, found that the LZM decreased the abundance of Tenericutes and the addition of probiotic yeast decreased the abundance of Tenericutes in pig’s feces. Tenericutes was thought to be associated with inflammation responses and was found in diet-induced obese mice [35] and obese Göttingen pigs’ fecal [36]. The present study showed that, anti-inflammatory cytokines IL-10 in serum increased on day 7 of lactation and had a tendency to increase on the farrowing day in the LZM treatments. Consistent with these findings, our previous study found that LZMA increased IL-10 in fecal on day 7 of sow lactation [18], and increased serum immunoglobulin M (IgM), immunoglobulin A (IgA), and milk IgA of sow [19]. These results showed that lysozyme does promote the expression of animal anti-inflammatory factors. This is consistent with the results reported in previous studies which found that lysozyme is involved in inflammatory response modulation [37,38].

Early study conducted in vitro culture of sow vaginal microorganisms by sterile guarded swabs method, found that the dominant bacterial genera were *Streptococcus* spp., *Escherichia coli*, *Staphylococcus* spp., *Corynebacterium* spp., *Micrococcus* spp. and *Actinobacillus* spp. [39]. High-throughput pyrosequencing of 16S rRNA gene results showed that at the genus level, *Bacillus*, *Paenibacillus*, *Alkaliphilus* and *Cronobacter* were the most abundant bacterial genera in healthy sows [26]. In this study, it was found that the most abundant bacterial genera in sows’ vagina were *Escherichia coli*, *Streptococcus*, *Enterococcus*, *Bacillus*, *Clostridium_sensu_stricto_1*, *Staphylococcus*, *Acinetobacter*, *Lactobacillus* and *Proteus*. Previous study found that the abundance of *Escherichia-Shigella*, *Bacteroides*, *Fusobacterium* and *Clostridium_sensu_stricto_1* in sows with endometritis was higher than healthy sows [26]. This indicates that these bacterial genera may be pathogenic bacteria in the vagina and thus have a higher abundance in animals which have vaginal diseases. *Clostridium sensu stricto* was reported to exhibit mucinase activity and can consume mucus-derived saccharides as energy sources [40]. The microbe-mediated mucin utilization would subsequently increase the production of short-chain fatty acids (SCFA) causing the host to respond with increased production, subsequently thickening the inner mucus layer. It is probable that an increase in the thickness of the inner mucus layer would delay pathogen adherence [38]. However, it was reported that *Clostridium_sensu_stricto_1* belongs to C. septicum (they shared > 97% sequence identity), since it induced cellulitis in turkey which is considered to be primarily caused by C. septicum [41]. During parturition, sows are more susceptible to infestation by pathogenic bacteria due to changes in the external environment and their own physiological state, which in sequence leads to vaginal inflammation and disease [42]. In present study, LZM treatments increased vaginal relative abundances of *Enterococcus* on the farrowing day, which was considered to be a probiotics microorganism that improves the reproductive performance of sows [43]. LZMA treatment decreased relative abundance of bacteria *Burkholderia-Paraburkholderia* and *Streptococcus* which contains multiple pathogen strains and also has proinflammatory properties [44]. LZMB treatment decreased relative abundance of *Escherichia-Shigella* and increased *Lactobacillus* in sow vagina in this study. *Lactobacilli*, known as an inhibitor of many vaginal pathogens of female animal [45,46]. *Lactobacillus* can acidify the vaginal environment by producing lactic acid and lower the vaginal pH to prevent the infection of microorganisms outside the vagina [47]. This not only ensures the relative stability of the microorganisms in the vagina, but also plays an important role in maintaining vaginal health [45,47]. In addition, the vaginal bacteria such as *Atopobium*, *Streptococcus*, *Enterococcus*, *Staphylococcus*, *Megasphaera*, *Bacillus* and other bacteria had the ability to produce lactic acid [48].

Interestingly, the vaginal microbiota of sows from parturition to lactation also changed at the genus level. Compared with the day of farrowing, the abundance of *Clostridium_sensu_stricto_1* in the vagina significantly decreased and *Enterococcus* significantly increased on day 7 of lactation. The abundance of *Bacillus* in the LZMB treatment significantly increased compared with the day of farrowing. The decrease of *Clostridium_sensu_stricto_1* and increase of *Enterococcus* and *Bacillus* bacteria are helpful to maintain the physiological state of the sow’s vagina and prevent the infection of pathogenic bacteria [26,48]. The increase of *Enterococcus* and *Bacillus* in the treatment also indicated that the addition of lysozyme could ameliorate the bacterial community structure of the vaginal flora of sows after farrowing.

In our previous study, it was found that adding lysozyme in sows’ (same batch of sows with the present study) diets increased sow average daily feed intake during the lactation, shortened the days of weaning-to-estrus interval, and decreased the stillborn number and the diarrhea rate of offspring [19]. However, no differences were observed between treatments in terms of the number of total born, born alive and weakling piglets, and neonatal weight [19]. It was also found that the typical changes in sows’ feces to the lysozyme supplementation were an increase in *Lactobacillus* genera and a decrease in Romboutsia, Spirochaetes, Actinobacteria and Tenericutes phylum [18]. In this study, correlation analysis revealed significant positive associations between vaginal and fecal *Desulfovibrio* and sow productive performance. The relative abundances of *Bacillus* in sows’ feces were positively corrected with neonatal weight. Study found that the fecal microbiota varied between high or low stillbirth rates in sows [49]. Similar to our findings, previous studies found that the gestation sow supplementation with probiotics which containing *Bacillus licheniformis* and *Bacillus subtilis spores* [50], or *Bacillus subtilis C-3102* [51] enhanced health status and reproductive performance of sow. In contrast with our findings, previous studies found that *Desulfiovibrio* was associate to higher gut inflammatory state in humans [52] and correlated with higher incidence of PEDV in piglets [53]. *Desulfovibrio*, being considered as obligate anaerobic organisms, belonging to the class of deltaproteobacteria, is a widely studied genus among the sulfate-reducing microorganism. *Desulfovibrio* can couple oxidation of a variety of electron donors, such as lactate or pyruvate, to the reduction of sulfate [54]. It can also reduce metal ions such as Fe(III) [55] and Cr(VI) [56]. Moreover, some *Desulfovibrio* strains have the ability to ferment organic compounds such as pyruvate [57]. These functions may be beneficial to the sow’s metabolism, and help understand why there is a positive correlation between *Desulfovibrio* and the sow’s total born and alive at birth.

## 5. Conclusions

In conclusion, these results suggested that lysozyme diet supplementation during the perinatal period of sow are beneficial to maintenance the vaginal microbial flora. The beneficial improvement of sow reproductive performance was associated with the alternations of vaginal and fecal microbiota and immune function.

## Figures and Tables

**Table 1:** Effect of lysozyme on serum cytokines of sow.

Item	Time		Treatment		*p*-Value	FDR
		CON.	LZMA	LZMB		
IL-6 (ng/l)	d 0	58.40 ± 7.12	56.48 ± 9.86	56.08 ± 9.93	0.90	0.93
	d 7	67.09 ± 3.41	63.88 ± 3.12	63.58 ± 3.58	0.23	0.35
IL-10 (ng/l)	d 0	250.78 ± 20.39	280.4 ± 14.75	274.06 ± 20.17	0.08	0.09
	d 7	266.67 ± 10.60 ^b^	287.36 ± 8.48 ^a^	284.09 ± 12.27 ^a^	0.02	0.04
TNF-α (ng/l)	d 0	122.68 ± 10.32	119.54 ± 9.21	121.1 ± 10.67	0.86	0.97
	d 7	123.38 ± 15.29	121.65 ± 18.77	122.54 ± 14.38	0.98	0.99

CON. = control diet, LZMA = control diet + lysozyme 150 mg/kg, LZMB = control diet + lysozyme 300 mg/kg, IL-6 = interleukin-6, IL-10 = interleukin-10, TNF-α = tumor necrosis factor-α. Values are mean ± SD (n = 8). d 0 = day of farrowing, d 7 = day 7 of lactation. ^a,b^ Within a row, means with different superscripts are different (*p* < 0.05).

**Table 2:** The relative abundances at phyla level (%, >0.1% in at least one sample) and genera level (%, >0.3% in at least one sample, show only significant differences here) in vaginal sample of feeding sows with lysozyme.

Phyla	Genera	Treatment			*p*-Value	FDR
		CON.	LZMA	LZMB		
**d 0**	**d 0**					
Firmicutes		52.77 ± 9.08	45.10 ± 6.94	41.20 ± 6.56	0.56	0.69
	*Streptococcus*	18.65 ± 7.15 ^a^	6.37 ± 1.35 ^b^	5.05 ± 1.13 ^b^	0.01	0.02
	*Enterococcus*	4.67 ± 1.16 ^b^	12.19 ± 4.67 ^a^	8.88 ± 2.32 ^a^	0.05	0.06
	*Bacillus*	4.22 ± 2.29 ^a^	2.66 ± 0.27 ^b^	1.19 ± 0.19 ^b^	0.03	0.04
	*Lachnospiraceae_XPB1014_group*	0.59 ± 0.10 ^b^	0.81 ± 0.15 ^ab^*	1.17 ± 0.25 ^a^	0.04	0.05
	*Lactobacillus*	0.46 ± 0.20 ^b^	0.89 ± 0.25 ^ab^*	2.11 ± 0.76 ^a^*	0.04	0.05
	*Oscillospira*	0.34 ± 0.04 ^a^*	0.23 ± 0.02 ^b^*	0.22 ± 0.03 ^b^*	0.03	0.04
	*Family_XIII_AD3011_group*	0.31 ± 0.04 ^a^*	0.17 ± 0.02 ^b^*	0.24 ± 0.03 ^b^*	0.04	0.05
	*Lachnospiraceae_AC2044_group*	0.28 ± 0.04 ^b^	0.23 ± 0.02 ^b^	0.39 ± 0.05 ^a^	0.03	0.04
Proteobacteria		42.88 ± 0.09	49.87 ± 6.7	54.19 ± 6.85	0.59	0.71
	*Acinetobacter*	1.09 ± 0.51 ^b^	4.28 ± 0.81 ^a^	4.53 ± 0.05 ^a^	<0.05	<0.05
	*Burkholderia-Paraburkholderia*	0.31 ± 0.27 ^a^	0.04 ± 0.01 ^b^	0.24 ± 0.09 ^a^	0.04	0.04
Bacteroidetes		2.12 ± 0.11 ^b^	3.06 ± 0.43 ^a^*	2.36 ± 0.37 ^ab^*	0.03	0.04
Tenericutes		1.16 ± 0.08	1.04 ± 0.10 *	1.14 ± 0.11 *	0.87	0.95
Verrucomicrobia		0.32 ± 0.06	0.24 ± 0.01	0.19 ± 0.01	0.61	0.76
Spirochaetes		0.21 ± 0.04 *	0.13 ± 0.001	0.23 ± 0.06 *	0.58	0.68
Actinobacteria		0.17 ± 0.001	0.21 ± 0.001	0.14 ± 0.01	0.69	0.81
Euryarchaeota		0.14 ± 0.001 *	0.10 ± 0.001 *	0.33 ± 0.11 *	0.64	0.79
d 7						
Firmicutes		43.81 ± 2.08	50.31 ± 6.84	46.58 ± 3.49	0.61	0.75
	*Streptococcus*	11.84 ± 2.21 ^b^	20.32 ± 4. 41 ^a^*	14.20 ± 1.22 ^ab^*	0.04	0.05
	*Ruminococcaceae_NK4A214_group*	1.37 ± 0.24 ^a^	0.85 ± 0.21 ^b^	0.98 ± 0.27 ^ab^	0.04	0.05
	*Ruminococcaceae_UCG-002*	1.13 ± 0.26 ^a^	0.53 ± 0.08 ^b^	0.62 ± 0.10 ^b^	0.03	0.04
	*Ruminococcaceae_UCG-005*	0.90 ± 0.12 ^a^	0.29 ± 0.05 ^b^	0.55 ± 0.10 ^b^	<0.05	<0.05
	*Terrisporobacter*	0.63 ± 0.18 ^a^	0.39 ± 0.18 ^b^	0.74 ± 0.05 ^a^	0.04	0.04
	*Lachnospiraceae_XPB1014_group*	0.55 ± 0.10	0.22 ± 0.04	0.54 ± 0.16	0.05	0.05
	*Christensenellaceae_R-7_group*	0.37 ± 0.03	0.24 ± 0.04	0.29 ± 0.05	0.05	0.06
	*Lactobacillus*	0.94 ± 0.46 ^a^	0.31 ± 0.21 ^b^	1.07 ± 0.33 ^a^	0.04	0.05
	*Family_XIII_AD3011_group*	0.22 ± 0.02 ^a^	0.09 ± 0.02 ^b^	0.13 ± 0.02 ^b^	<0.05	<0.05
Proteobacteria		52.96 ± 2.14	47.59 ± 7.14	50.77 ± 3.39	0.73	0.86
	*Escherichia-Shigella*	36.58 ± 6.16 ^a^	37.98 ± 8.14 ^a^	24.60 ± 3.34 ^b^	0.03	0.04
	*Acinetobacter*	7.31 ± 2.28 ^b^*	5.03 ± 1.62 ^b^	18.15 ± 2.31 ^a^*	<0.05	<0.05
Bacteroidetes		1.58 ± 0.45	0.79 ± 0.12	1.52 ± 0.22	0.15	0.38
Tenericutes		0.96 ± 0.16 ^a^	0.28 ± 0.06 ^b^	0.30 ± 0.05 ^b^	<0.05	<0.05
Verrucomicrobia		0.20 ± 0.003	0.20 ± 0.001	0.17 ± 0.001	0.69	0.79
Spirochaetes		0.11 ± 0.006	0.10 ± 0.001	0.09 ± 0.001	0.85	0.91
Actinobacteria		0.20 ± 0.01	0.37 ± 0.02 *	0.29 ± 0.05 *	0.07	0.08
Euryarchaeota		0.01 ± 0.01	0.01 ± 0.02	0.01 ± 0.001	0.87	0.94

Data are expressed as mean ± SD. Sows were regarded as the experimental units, n = 6 for each treatment. CON. = control diet, LZMA = control diet + lysozyme 150 mg/kg, LZMB = control diet + lysozyme 300 mg/kg. d 0 = on the day of farrowing, d 7 = day 7 of lactation. ^a,b^ Within a row, means with different superscripts are different (*p* < 0.05). * Within a column in the same index at different day, means with asterisk denotes different (*p* < 0.05).

**Table 3:** Effects of lysozyme on microbiota alpha diversity index of sow vagina.

Item	Time		Treatment		*p*-Value		
		CON.	LZMA	LZMB	Diet	Time	Diet*Time
Observed species	d 0	821.50 ± 35.67 *	931.33 ± 38.94 *	792.50 ± 32.59 *	0.89	<0.01	<0.01
	d 7	734.00 ± 55.27 ^a^	586.67 ± 34.27 ^b^	651.83 ± 43.07 ^b^			
Chao 1	d 0	919.63 ± 42.72	1009.63 ± 43.39 *	890.06 ± 37.91 *	0.81	<0.01	<0.01
	d 7	841.68 ± 62.32 ^a^	663.14 ± 40.74 ^b^	779.90 ± 51.05 ^b^			
Shannon	d 0	4.14 ± 0.42	4.29 ± 0.20	3.84 ± 0.37	0.37	0.77	0.15
	d 7	4.01 ± 0.22	3.45 ± 0.28	4.17 ± 0.14			

Data are expressed as mean ± SD. Sows were regarded as the experimental units, n = 6 for each treatment. CON. = control diet, LZMA = control diet + lysozyme 150 mg/kg, LZMB = control diet + lysozyme 300 mg/kg. d 0 = day of farrowing, d 7 = day 7 of lactation. ^a,b^ Within a row, means with different superscripts are different (*p* < 0.05). * Within a column in the same index at different day, means with asterisk denotes different (*p* < 0.05).

**Table 4:** Spearman correlations between the vagina or rectum microbiota at genus level and sow performance.

	*Escherichia.Shigella*	*Streptococcus*	*Clostridium_sensu_stricto_1*	*Lactobacillus*	*Ruminococcaceae_UCG.014*	*Terrisporobacter*	*Desulfovibrio*	*Bacillus*
Correlations between the vagina microbiota and sow performance								
Total born	−0.083	−0.004	0.140	0.173	0.003	0.081	0.273	−0.107
Born alive	−0.069	−0.169	0.254	0.147	−0.136	0.159	0.309 *	−0.073
Stillborn	0.078	0.214	−0.131	−0.116	0.165	−0.154	−0.011	−0.001
Neonatal weight	0.236	−0.162	0.116	0.152	−0.098	0.146	0.181	−0.141
Correlations between the rectum microbiota and sow performance								
Total born	−0.131	−0.091	0.177	0.089	−0.177	0.218	0.357 **	−0.072
Born alive	−0.070	−0.255	−0.355 **	0.064	−0.376 **	0.375 **	0.446 **	0.069
Stillborn	−0.078	0.214	−0.208	0.007	0.284	−0.281	−0.166	−0.235
Neonatal weight	−0.090	0.148	−0.134	0.096	−0.201	−0.017	−0.143	0.368 **

* denotes *p* < 0.1 and ** denotes *p* < 0.05.

## Data Availability

Data is contained within the article or supplementary material. For more detailed data about this study please request from the corresponding author.

## Supplementary Materials

The following are available online at https://www.mdpi.com/2076-2615/11/3/593/s1, Figure S1: Heatmap of the spearman r correlations between the vaginal microbiota (phylum level) significantly modified by metabolic parameters of sow, Figure S2: Venn diagrams were generated to compare OTUs between the microbiota of vagina and rectum of different treatments on the day of farrowing (d 0) and day 7 of lactation (d7), Table S1: Average raw reads, effective tags and OTUs of vaginal microbial community from d 1 to d 7 of lactation in vagina, Table S2: The relative abundances at genera level (%, > 0.3% in at least one sample, show not significant differences here) in vaginal sample of feeding sows with lysozyme.
