# Supplementary figures and images for: Responses of Vaginal Microbiota to Dietary Supplementation with Lysozyme and its Relationship with Rectal Microbiota and Sow Performance from Late Gestation to Early Lactation
Source: Animals (Basel). 2021 Feb 24;11(3):593. doi: 10.3390/ani11030593 (PMC7996156; doi:10.3390/ani11030593)

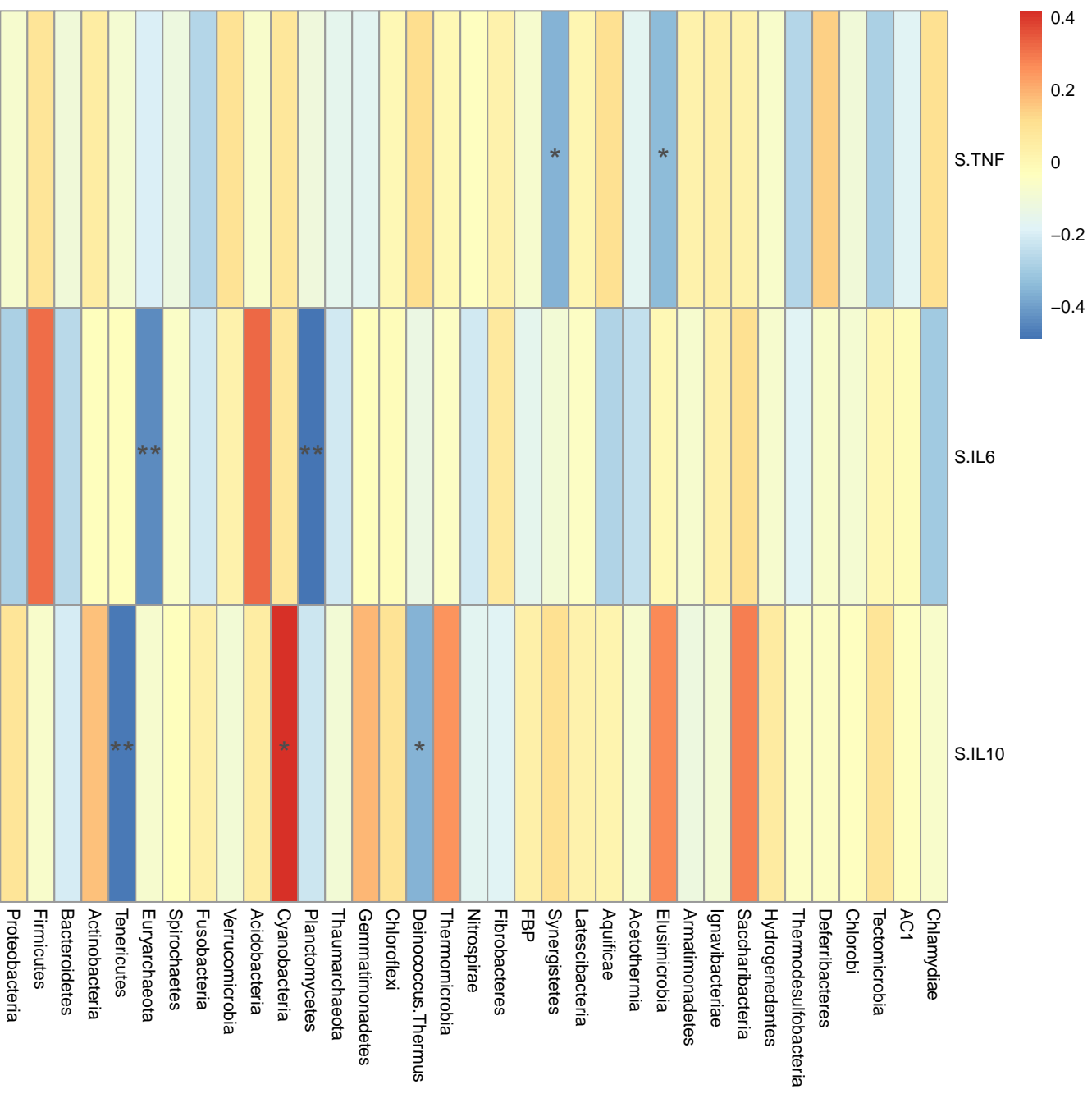

Supplement: Supplementary file 1 [file animals-11-00593-s001.zip › supplementary/Figure S1.pdf]

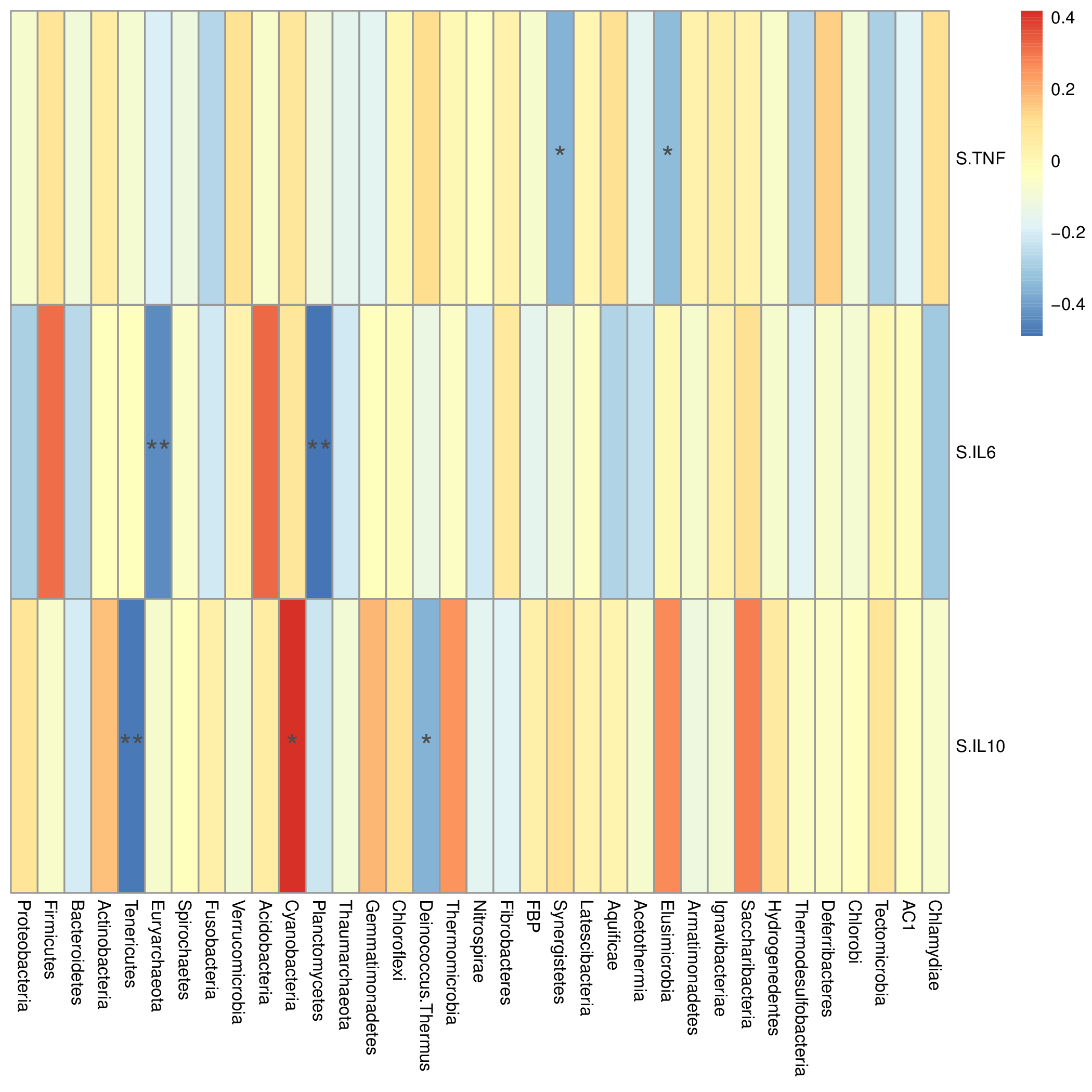

Supplement: Supplementary file 1 [file animals-11-00593-s001.zip › supplementary/Figure S1.png]

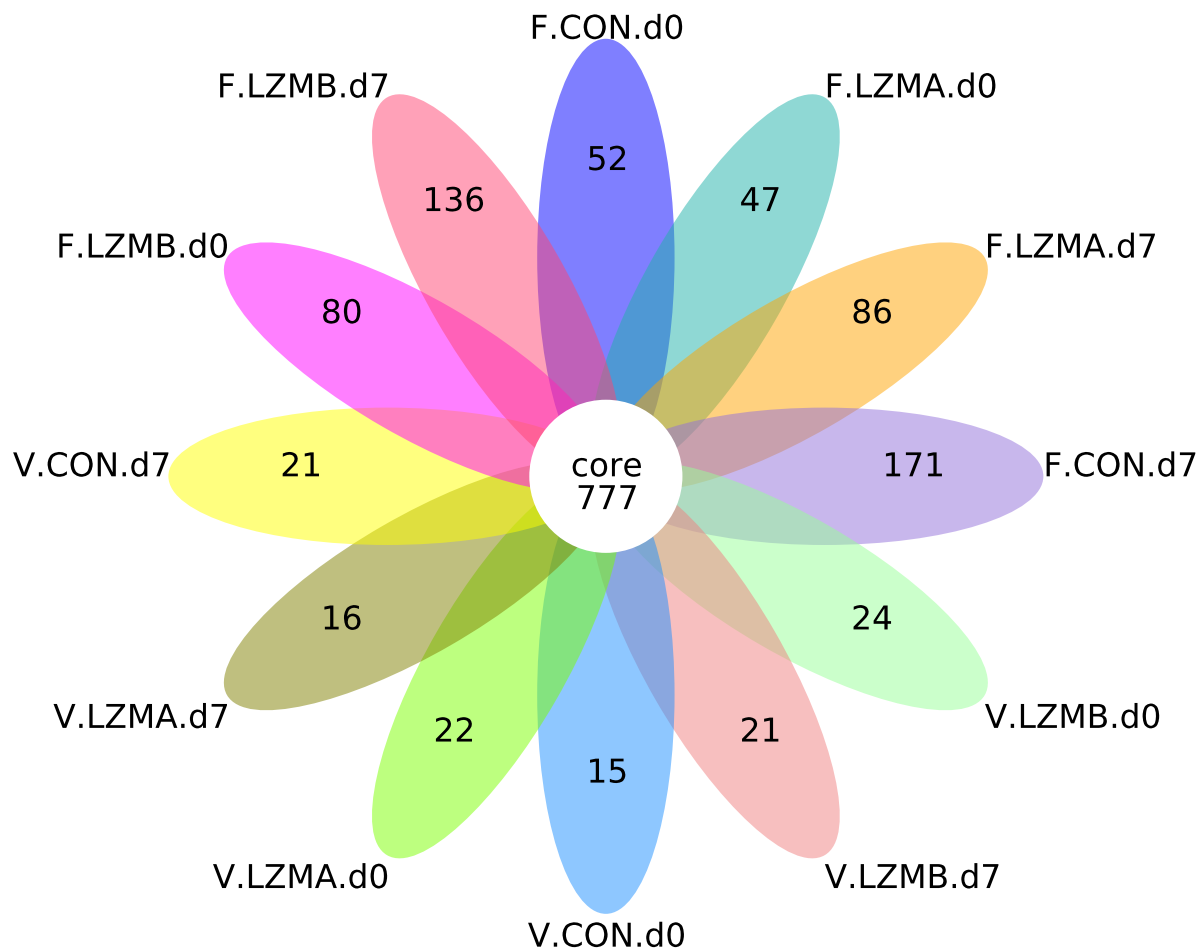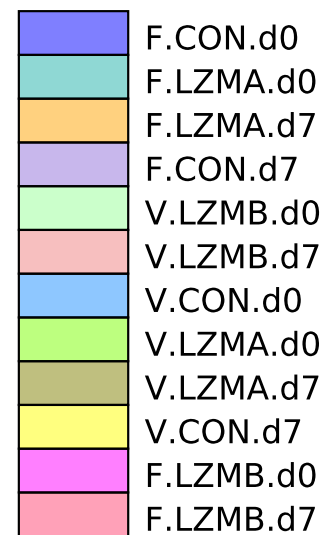

Supplement: Supplementary file 1 [file animals-11-00593-s001.zip › supplementary/Figure S2.pdf]

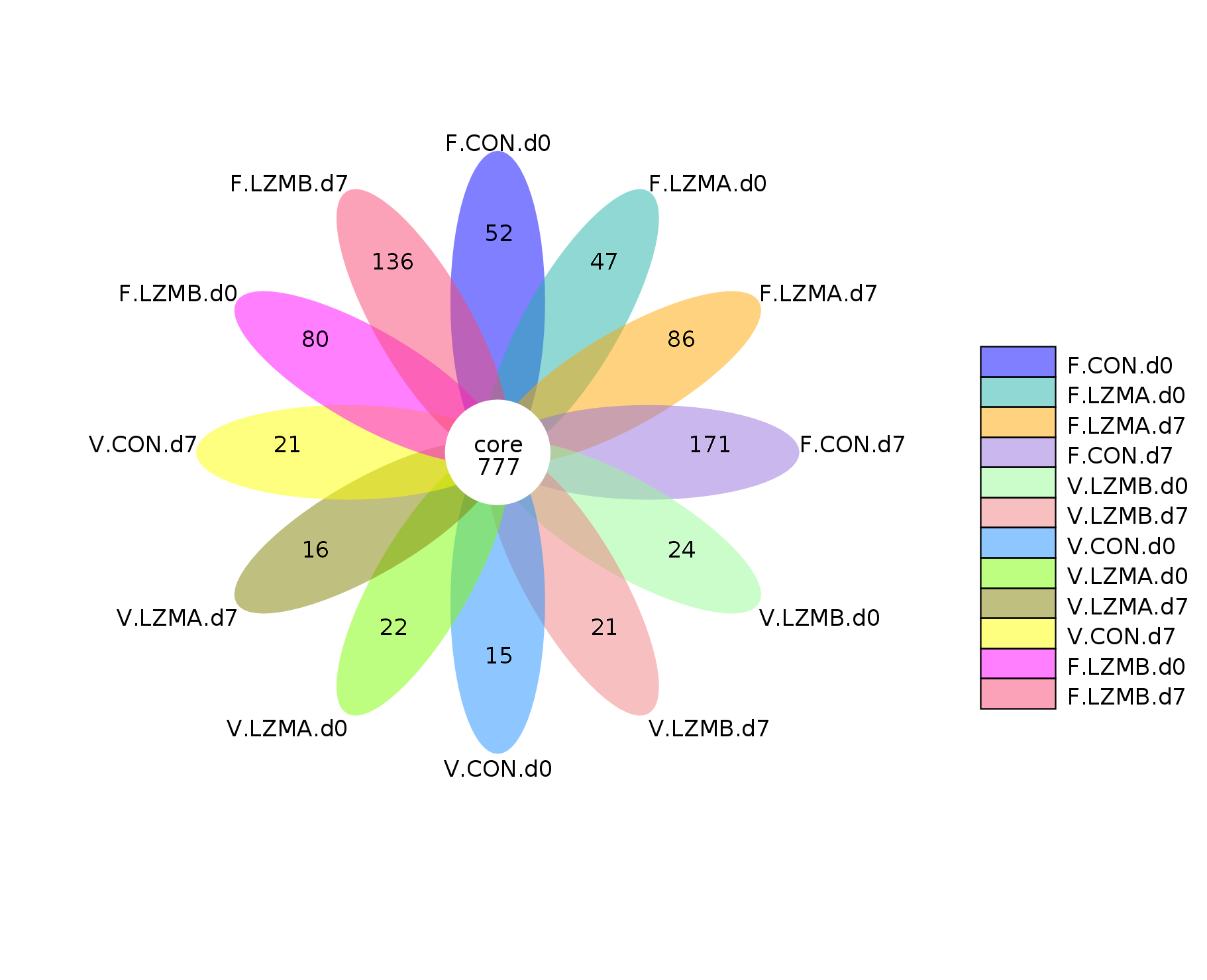

Supplement: Supplementary file 1 [file animals-11-00593-s001.zip › supplementary/Figure S2.png]
